# A novel nomogram to predict lymph node metastasis in cT1 non-small-cell lung cancer based on PET/CT and peripheral blood cell parameters

**DOI:** 10.1186/s12890-023-02341-7

**Published:** 2023-01-30

**Authors:** Bohua Wei, Xin Jin, Gaojun Lu, Teng Zhao, Hanjiang Xue, Yi Zhang

**Affiliations:** 1 Department of Thoracic Surgery, Xuanwu Hospital, Capital Medical University, No. 45 Changchun Street, Beijing, China; 2 Laboratory of Respiratory Disease and Thoracic Surgery, KU Leuven, 3000 Leuven, Belgium

**Keywords:** Non-small-cell lung cancer, Maximum standard uptake value, Consolidation tumor ratio, Platelet to lymphocyte ratio, Nomogram

## Abstract

**Background:**

Accurately evaluating the lymph node status preoperatively is critical in determining the appropriate treatment plan for non-small-cell lung cancer (NSCLC) patients. This study aimed to construct a novel nomogram to predict the probability of lymph node metastasis in clinical T1 stage patients based on non-invasive and easily accessible indicators.

**Methods:**

From October 2019 to June 2022, the data of 84 consecutive cT1 NSCLC patients who had undergone PET/CT examination within 30 days before surgery were retrospectively collected. Univariate and multivariate logistic regression analyses were performed to identify the risk factors of lymph node metastasis. A nomogram based on these predictors was constructed. The area under the receiver operating characteristic (ROC) curve and the calibration curve was used for assessment. Besides, the model was confirmed by bootstrap resampling.

**Results:**

Four predictors (tumor SUVmax value, lymph node SUVmax value, consolidation tumor ratio and platelet to lymphocyte ratio) were identified and entered into the nomogram. The model indicated certain discrimination, with an area under ROC curve of 0.921(95%CI 0.866–0.977). The calibration curve showed good concordance between the predicted and actual possibility of lymph node metastasis.

**Conclusions:**

This nomogram was practical and effective in predicting lymph node metastasis for patients with cT1 NSCLC. It could provide treatment recommendations to clinicians.

## Introduction

Lung cancer is the leading cause of cancer-related death among malignant tumors [1]. It is typically classified as non-small-cell lung cancer (NSCLC) and small-cell lung cancer (SCLC) according to clinical practice. NSCLC accounts for more than 85% of all cases. Lymph node (LN) staging is crucial in determining the therapeutic strategy and prognosis of lung cancer patients [2]. Although lobectomy is still the gold standard for early-stage NSCLC patients, sub-lobar resection has been shown to achieve similar effects [3, 4]. Besides, stereotactic ablative radiotherapy (SABR) can be an option for stage I patients especially in the elderly [5, 6]. However, for stage N1 and N2 patients, lobectomy with systemic mediastinal lymph node dissection (SLD) is strongly recommended. Hence, accurately predicting lymph node status before pathological confirmation is crucial to clinicians especially in cT1 patients.

Invasive procedures such as endobronchial ultrasound transbronchial needle aspiration (EBUS-TBNA) or mediastinoscopy definitely can achieve high sensitivity and specificity [7, 8]. Nevertheless, their additional trauma, expense and complications cannot be ignored. Thus, in the present study, we attempted to find a method to predict lymph node status based on non-invasive and easily accessible indicators.

18F-fluorodeoxyglucose positron emission tomography/computed tomography (PET/CT) is a common method to evaluate lymph node status preoperatively in lung cancer patients. Maximum standard uptake value (SUVmax) is the most commonly used index. Elevated uptake of lymph nodes usually suggestive of metastasis. However, the accuracy could not be completely satisfying [9]. Besides, several studies have confirmed higher SUVmax of the primary lesion was related to lymph node metastasis [10–13].

Consolidation tumor ratio (CTR) is one of the crucial factors associated with lymph node metastasis. Tumors with more solid components are more likely to present with lymph node metastasis [12, 14, 15]. In addition, some inflammatory blood cell parameters such as platelet-lymphocyte ratio (PLR) and neutrophil–lymphocyte ratio (NLR) are useful in evaluating therapeutic effects and prognosis in NSCLC patients [16, 17]. Elevated NLR and PLR usually indicate higher invasion of the tumor. A significant difference was seen in the two parameters between patients with different N stages [18, 19].

Since its first application, nomogram has been accepted as a reliable tool to graphically depict the generating probability of a clinical event [20, 21]. In this study, we constructed a novel nomogram to predict lymph node status in cT1 NSCLC patients based on those aforementioned indicators.

## Methods

### Patients

We retrospectively enrolled 84 cT1 NSCLC patients who underwent lobectomy with systemic lymph node dissection in the thoracic surgery department of Xuanwu Hospital, Capital Medical University between October 2019 and June 2022. The inclusion criteria were as follows: (1) patients had undergone PET/CT in 30 days before surgical resection and no evidence of distant metastasis were found; (2) patients had completely peripheral blood cell parameters within 5 days before operation; (3) patients’ necessary clinicopathological data were complete. And we excluded these patients: (1) patients had undergone chemotherapy, radiotherapy, targeted therapy or immunotherapy before surgery; (2) patients had clinical evidence of acute infection; (3) patients had a history of other malignancies; (4) patients had a history of hematological or immune system disorders. (5) patients with possible distant metastasis suggested by PET/CT. Tumor staging was determined according to the eighth edition of the TNM classification for NSCLC [22]. Patients with pathologically confirmed pN1 or pN2 were defined as pN+. Otherwise, they were defined as pN0.

This study was performed in accordance with relevant principles outlined in the Declaration of Helsinki and it was approved by the Xuanwu Hospital Ethic committee. Written informed content was obtained from all enrolled patients.

### Chest CT scan

Chest CT scans were examined with a window level of − 700 Hounsfield Unit (HU) and a window width of 1500 HU as the lung window. The mediastinal window was defined as a window level of 40 HU and a window width of 350 HU. CTR was calculated by the maximum diameter of the lesion on the mediastinal window divided by the diameter on the lung window.

### PET/CT examination

PET/CT examination was conducted in all patients with an integrated PET/CT scanner (Biograph-16, Siemens, Germany). Patients fasted for at least 6 h before the examination and then would be injected with 18F-fluorodeoxyglucose. After 40 min, images were obtained from the plane of skull to the groin level. The SUVmax of the primary tumor and suspicious lymph node were determined by drawing a region of interest around it.

### Peripheral blood cell parameters

Blood samples were drawn in a fasting state and stored in collection tubes containing ethylene diamine tetraacetic acid (EDTA). The complete blood count test was analyzed by Sysmex XE-5000 automated hematology analyzer. PLR was defined as platelet count/lymphocyte count. NLR was defined as neutrophil count/lymphocyte count.

### Surgical procedures

All patients underwent lobectomy and systemic lymph node dissection by video-assisted thoracoscopic surgery (VATS). Systemic LN dissection was performed according to the European Society of Thoracic Surgeons guidelines [23]. At least 3 mediastinal stations including subcarinal station were excised. The minimal number of dissected LNs was 6.

### Statistical analysis

IBM SPSS (version 23.0) and R software (version 4.0.3) were used for statistical analysis. Continuous variables were displayed by mean and standard deviation and analyzed with a t-test. Categorical variables were displayed by numbers and percentages and analyzed with χ^2^ or Fisher’s exact tests. Univariate and multivariate logistic regression analyses were performed to identify the potential factors related to lymph node metastasis of cT1 NSCLC patients. An odds ratio (OR) with a 95% confidence interval (CI) was used to estimate correlation strength. Then the nomogram was established based on multivariable analysis. The performance of it was assessed by discrimination and calibration. The model’s discriminative ability was determined by the area under receiver operating characteristic (ROC) curve ranging from 0.5 (no discrimination) to 1 (perfect discrimination). It was calibrated by a visual plot comparing the predicted and actual probability of lymph node metastasis. Besides, the nomogram was subjected to a 500 bootstraps resamplings for internal validation [24]. A two-sided *p* < 0.05 was considered statistically significant among all analysis methods.

## Results

### Basic data and univariate analysis

A total of 84 patients consisting of 40 males and 44 females were enrolled in the study. The overall incidence of lymph node metastasis was 25.0% (21/84). Among these patients, 3 patients had N1 LN metastasis only. 7 patients had skip N2 LN metastasis and 11 had N1 and N2 LN involvement. The specific characteristics of the enrolled patients were shown in Table 1. Compared with pN0 patients, pN+ patients had larger tumor size (2.27 ± 0.33 cm vs. 2.06 ± 0.49 cm), higher tumor SUVmax value (11.54 ± 8.65 vs. 4.42 ± 4.32), higher lymph node SUVmax value (4.69 ± 4.88 vs. 1.66 ± 3.58) and higher CTR (0.79 ± 0.13 vs. 0.40 ± 0.30) (*p* < 0.05). In terms of peripheral blood cell parameters, PLR (155.29 ± 48.98 vs. 115.65 ± 39.82) and NLR (2.40 ± 0.83 vs. 1.74 ± 0.97) were both significantly higher in pN + patients (*p* < 0.01).

**Table 1:** Patients’ basic data and univariate analysis

Characteristics	pN + (n = 21)	pN0 (n = 63)	t/χ^2^	*p*
Age	62.86 ± 11.01	61.48 ± 8.54	0.595	0.553
Sex			0.255	0.614
Male	9 (42.9%)	31 (49.2%)		
Female	12 (57.1%)	32 (50.8%)		
Smoking history			0.269	0.604
Yes	7 (33.3%)	25 (39.7%)		
No	14 (66.7%)	38 (60.3%)		
Tumor side			1.728	0.189
Left	5 (23.8%)	25 (39.7%)		
Right	16 (76.2%)	38 (60.3%)		
Histology			3.038	0.219
Adenocarcinoma	19 (90.4%)	60 (95.2%)		
Squamous cell carcinoma	1 (4.8%)	3 (4.8%)		
Other	1 (4.8%)	0		
Tumor size, cm	2.27 ± 0.33	2.06 ± 0.49	2.237	0.030*
Tumor SUVmax	11.54 ± 8.65	4.42 ± 4.32	3.625	0.001*
Lymph node SUVmax	4.69 ± 4.88	1.66 ± 3.58	2.625	0.014*
Consolidation tumor ratio	0.79 ± 0.13	0.40 ± 0.30	8.346	< 0.001*
PLR	155.29 ± 48.98	115.65 ± 39.82	3.725	< 0.001*
NLR	2.40 ± 0.83	1.74 ± 0.97	2.815	0.006*

SUVmax, maximum standard uptake value; CEA, carcinoembryonic antigen; PLR, platelet to lymphocyte ratio; NLR, neutrophil to lymphocyte ratio

*p < 0.05

### Multivariate analysis

Multivariate logistic regression analysis was conducted based on the risk factors picked up by univariate analysis (Table 2). The results showed that Tumor SUVmax (OR 1.154; 95%CI 1.003–1.329), lymph node SUVmax (OR 1.219; 95%CI 1.016–1.463), CTR (OR 973.847; 95%CI 5.396–175,746.732), and PLR (OR 1.026; 95%CI 1.004–1.048) were independent risk factors of lymph node metastasis.

**Table 2:** Multivariate analysis of characteristics related to lymph node metastasis

Characteristics	OR	95%CI	*P* value
Tumor size	1.855	0.208–16.560	0.580
Tumor SUVmax	1.154	1.003–1.329	0.046*
Lymph node SUVmax	1.219	1.016–1.463	0.033*
Consolidation tumor ratio	973.847	5.396–175,746.732	0.009*
PLR	1.026	1.004–1.048	0.020*
NLR	0.664	0.292–1.508	0.328

OR, odds ratio; CI, confidence interval; SUVmax, maximum standard uptake value; PLR, platelet to lymphocyte ratio; NLR, neutrophil to lymphocyte ratio

*p < 0.05

### The establishment and assessment of nomogram

**Fig. 1:**
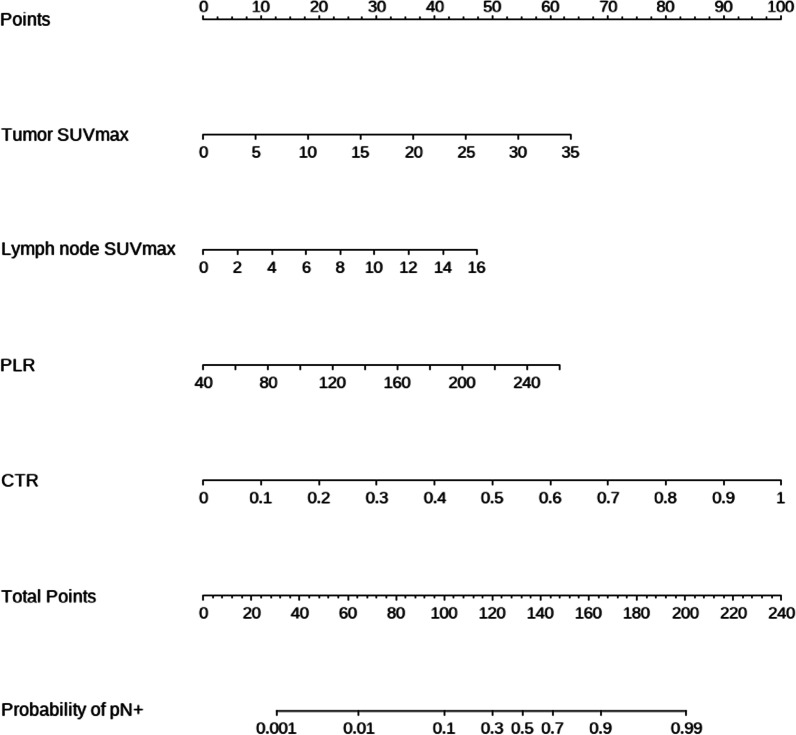
Nomogram for predicting the probability of lymph node metastasis in cT1 stage non-small-cell lung cancer. The value of each indicator was given a score on the point scale axis. The total score could be calculated by adding every single score. By projecting the total points to the probability of pN+ axis, we were able to estimate the probability of lymph node metastasis of the patients. SUVmax, maximum standard uptake value; PLR, platelet to lymphocyte ratio; CTR, consolidation tumor ratio

**Fig. 2:**
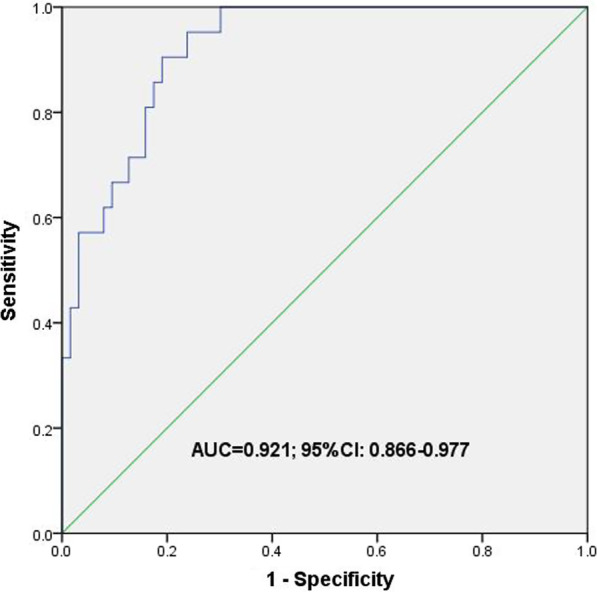
ROC curve of the nomogram in prediction of lymph node metastasis in cT1 NSCLC patients. ROC, receiver operating characteristic; AUC, area under curve; CI, confidence interval

**Fig. 3:**
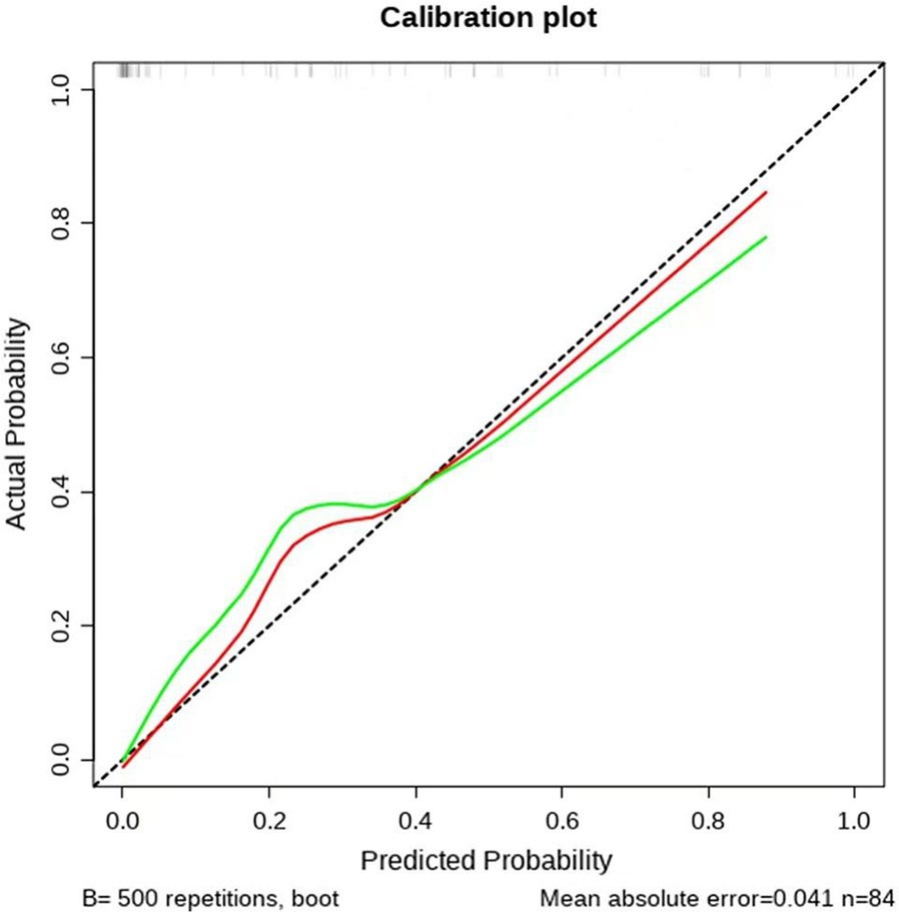
The calibration curve of the nomogram. The x axis represented the predicted probability, and the y axis represented the actual probability of lymph node metastasis. A perfect prediction would correspond to the black dashed line. The red and the green line respectively showed the apparent and bias corrected curve, which represented the performance of the nomogram

The four risk factors selected by multivariate analysis were used to establish the nomogram (Fig. 1). The area under ROC curve was 0.921 (95%CI 0.866–0.977) (Fig. 2), indicating certain discrimination ability. After 500 bootstrap self-sampling internal validation, the calibration curve of the model showed a relatively good concordance between the predicted and actual probability (Fig. 3).

## Discussion

In this retrospective study, we constructed and confirmed a novel nomogram to predict the incidence of lymph node metastasis in cT1 NSCLC patients based on non-invasive and easily accessible indicators. Primary tumor SUVmax value, lymph node SUVmax value, CTR and PLR were revealed as independent risk factors. The model demonstrated good discrimination and calibration. All the predictors applied in the model were specific continuous variables, which could minimize errors as far as possible. Besides, the length of the probability segment was relatively short between 0.1 to 0.9, which could better discriminate between cases with high and low-incidence of lymph node metastasis. Therefore, it might have potential application value for evaluating lymph node status preoperatively for cT1 NSCLC patients and could offer treatment guidance to clinicians.

Due to the importance of preoperative evaluation of LN status, several models based on PET/CT have been developed to predict LN metastasis [25–27]. Consistent with previous results, the SUVmax value of the primary tumor and the suspicious LN were identified as risk factors of LN metastasis [25, 26]. Zhao and colleagues found 0.61 as the optimal cut-off value of CTR for lymph node metastasis with high sensitivity and specificity [15]. Similarly, other cut-off values of CTR such as 0.8 or 0.62 were also reported associated with positive LN [12, 28]. However, in our view, it’s more pragmatic to apply the concrete CTR values in the model for the effects of measurement error could be reduced as much as possible.

Chronic inflammation serves an important role in tumor progression, invasion and metastasis. Generally speaking, neutrophils mainly play a role in promoting tumor invasion and metastasis by interacting with other immune cells to regulate innate and adaptive immunity, release angiogenic factors to promote tumor growth and release neutrophil extracellular traps (NETs) to inhibit the antitumor activity of NK cells and CD8+ lymphocytes [29, 30]. Platelets also mainly promote tumor invasion and metastasis by protecting tumor cells from shear forces and assault of NK cells and secreting growth factors to stimulate tumor cell proliferation to form micro metastasis foci. Meanwhile, platelet-derived growth factors help to open the capillary endothelium to accelerate tumor cell extravasation [31]. On the contrary, lymphocytes, especially CD8+ T cells are mainly responsible for combating external infection, clearing variant cells in the body, to exert inhibitory effects on tumor generation and progression. To our knowledge, few studies have attempted to incorporate peripheral blood cell parameters into the LN metastasis prediction model. Lv and colleagues compared the NLR between node-positive and node-negative patients but no difference was found [26]. Nevertheless, in the present study, we found NLR and PLR were both significantly higher in pN+ patients, consistent with the results of Chen and Wang [18, 19]. This may be mainly due to the higher proportion of N2 stage cases among patients presented with lymph nodes metastasis in our cohort. However, only PLR was identified as an independent predictor by multivariate analysis. Hence, studies with large sample size are needed to further study on this. Micropapillary and solid components have been proved to be related to LN metastasis [32, 33]. Incorporating this indeed further increased the diagnostic accuracy [11, 15]. However, obtaining frozen section (FS) sometimes could be difficult limited by the position of the lesion. Besides, determination of pathological subtype by FS is challenging for most pathologists, causing relatively low accuracy [34].

Systemic lymph node dissection is still the golden standard in lung cancer surgery. However, it’s associated with longer operative time, higher blood loss, higher incidence of postoperative complications and longer length of stay [35, 36]. Therefore, lobe-specific lymph node dissection options may be a better choice for patients with a low-incidence of lymph node metastasis predicted by the model [36, 37].

For stage IB-IIIA NSCLC patients, compared with surgery alone, neoadjuvant chemotherapy improves 5-year survival rate by 5% but appears to show no significant survival benefit compared with adjuvant chemotherapy [38, 39]. Recently, immune checkpoint inhibitors have profoundly changed the treatment paradigm for NSCLC patients. Theoretically, neoadjuvant immunotherapy could reach a better performance than adjuvant immunotherapy and it has been proven in preclinical animal models [40, 41]. Besides, immunotherapy is better tolerated in most patients and has minimal influence on surgery. Several studies have confirmed the safety and feasibility of preoperative immunotherapy [42, 43]. Thus, expert consensus indicates that preoperative use of neoadjuvant immunotherapy with or without platinum-based chemotherapy for patients with resectable stage IB‒IIIA NSCLC may be considered [44]. Since neoadjuvant therapy is generally not considered for stage IA patients while cT1 patients will upstage to at least stage IIB once exist of LN metastasis [22]. For this, our model may be useful to identify the cT1 patients with a-high incidence of LN involvement and fit for neoadjuvant therapy.

There were several limitations in our study. First, its retrospective and single-center nature therefore led to some inevitable bias. Second, the sample size was quite small compared with other studies. Therefore, we just simply divided the patients into two groups based on the presence or absence of lymph node metastasis. Besides, PET/CT scanning is not a routine examination for all NSCLC patients in our department. Thus, we could just review the subset of patients who underwent it. Moreover, though internal validation was performed to minimize the adverse effects and calibrate the model, data from other institutions are needed to test and optimize the nomogram for future use.

## Conclusions

Primary tumor SUVmax, lymph node SUVmax, CTR and PLR were identified as independent predictors of lymph node metastasis for patients with cT1 NSCLC. Based on these non-invasive and easily accessible indicators, we constructed and validated a novel nomogram. It offers a simple but effective means to assess lymph node status preoperatively and may guide clinical treatment.

## Data Availability

The datasets used and/or analyzed during the current study are available from the corresponding author on reasonable request.

## Abbreviations

NSCLC: Non-small-cell lung cancer
SCLC: Small-cell lung cancer
LN: Lymph node
SABR: Stereotactic ablative radiotherapy
SLD: Systemic mediastinal lymph node dissection
EBUS-TBNA: Endobronchial ultrasound transbronchial needle aspiration
PET/CT: Positron emission tomography/computed tomography
SUVmax: Maximum standard uptake value
CTR: Consolidation tumor ratio
PLR: Platelet–lymphocyte ratio
NLR: Neutrophil–lymphocyte ratio
EDTA: Ethylene diamine tetraacetic acid
VATS: Video-assisted thoracoscopic surgery
OR: Odds ratio
CI: Confidence interval
FS: Frozen section
